# Supplementary material for: Mendelian Randomisation Confirms the Role of Y-Chromosome Loss in Alzheimer’s Disease Aetiopathogenesis in Men
Source: Int J Mol Sci. 2023 Jan 4;24(2):898. doi: 10.3390/ijms24020898 (PMC9863537; doi:10.3390/ijms24020898)

Figure S8. Heatmap displaying Pearson correlation coefficients (R) for total tau, p-tau and CSF proteins measured with Olink Neurology (a) and Inflammation (b) panels. Olink NPX values were normalized (mean=0, SD=1) prior to computing R values.

a.

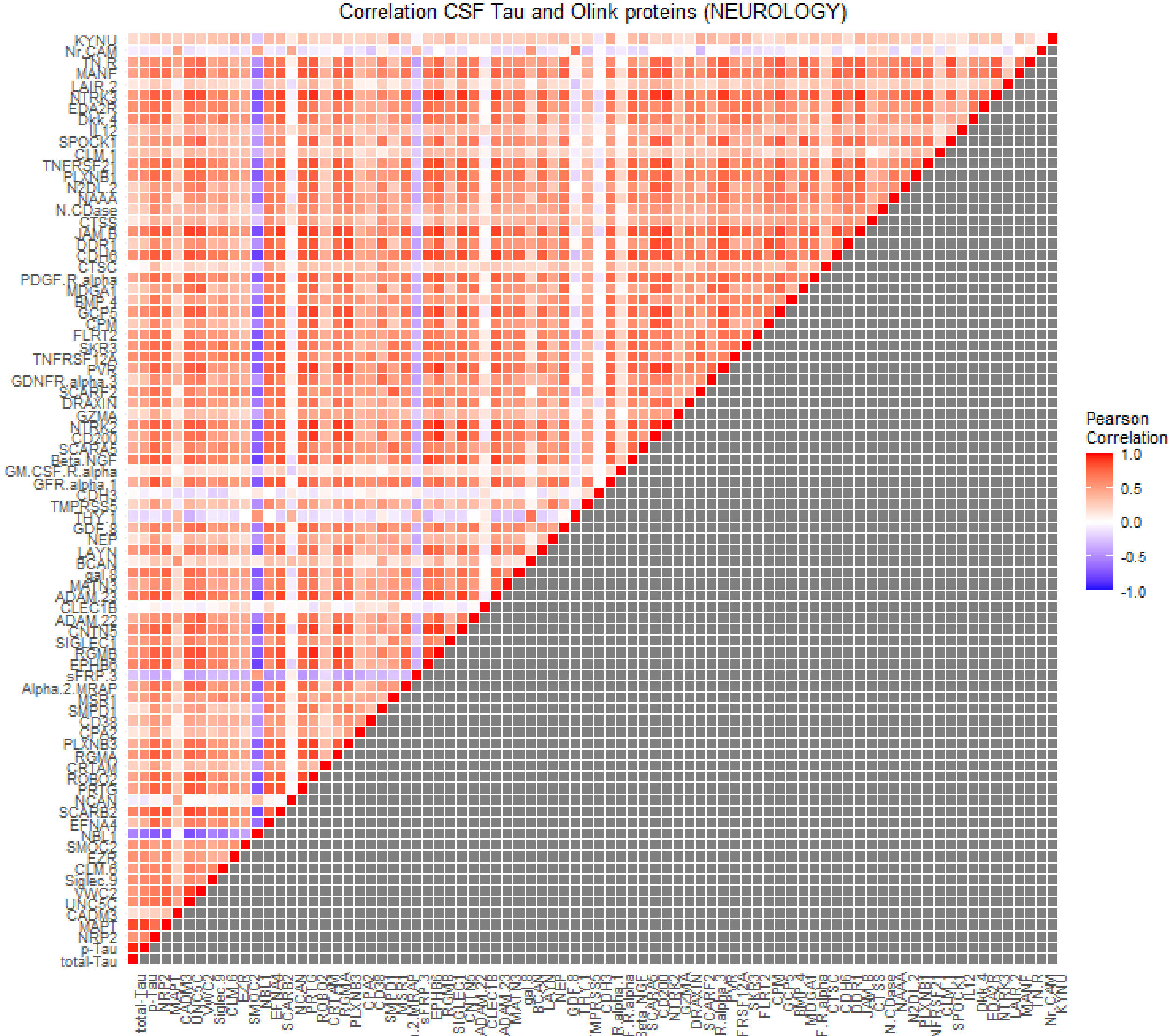

b.

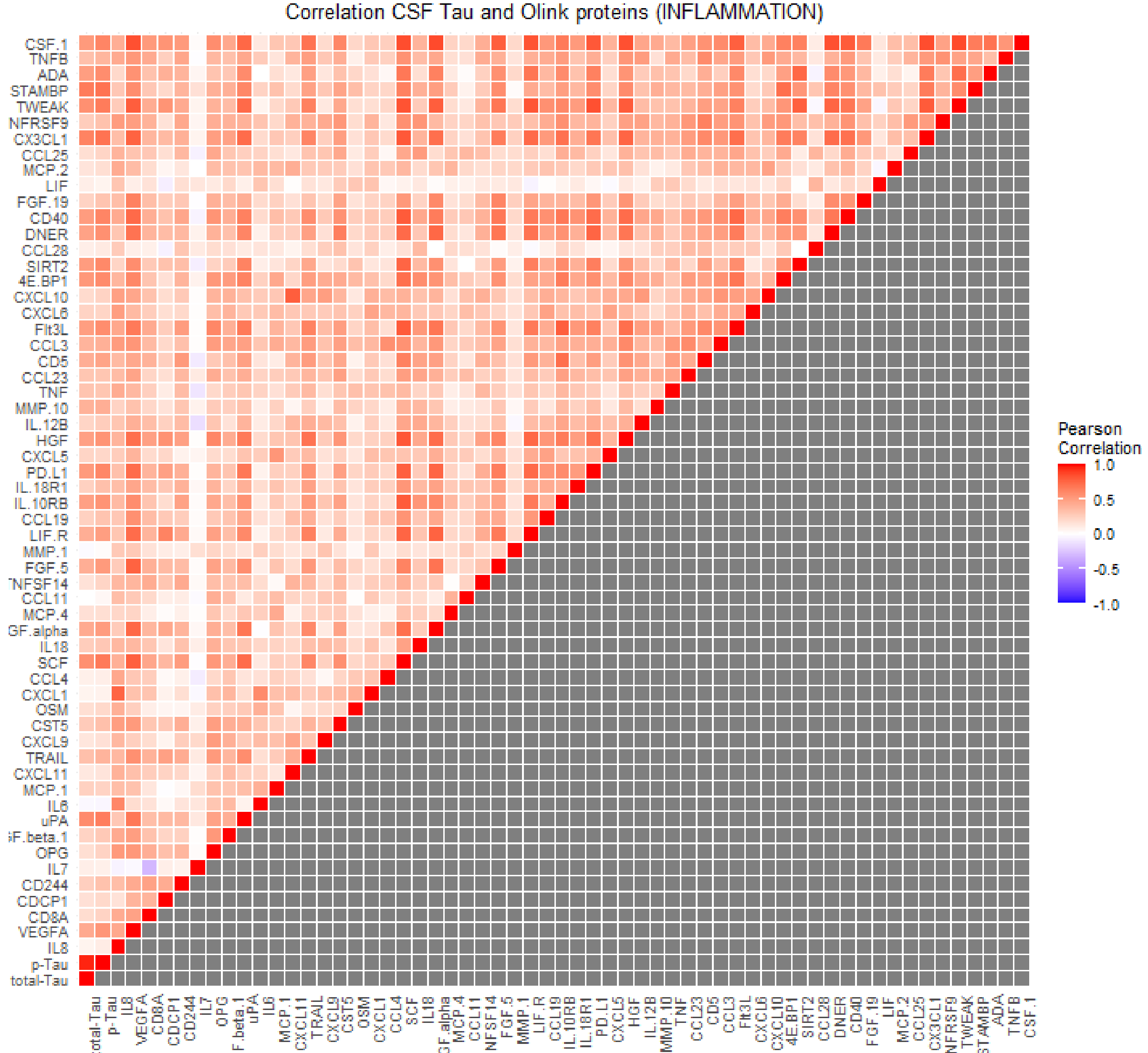

Supplement: Supplementary file 1 [file Figure_S8.pdf › Figure_S8.pdf]
