# Supplementary material for: Mendelian Randomisation Confirms the Role of Y-Chromosome Loss in Alzheimer’s Disease Aetiopathogenesis in Men
Source: Int J Mol Sci. 2023 Jan 4;24(2):898. doi: 10.3390/ijms24020898 (PMC9863537; doi:10.3390/ijms24020898)

Figure S1. Sex check and representation of mLRR-Y and PAR1-Bdev parameters in the GR@ACE/DEGESCO cohort. a. Plot of mean log R Ratio for chromosome X (mLRR-X) and chromosome Y (mLRR-Y). The top left corner represents genetically normal XY men, with an observable degradation of mLRR-y values representing individuals with varying degrees of mLOY. Bottom right corner represents genetically normal women (XX) and top right corner Klineffelter (XXY) individuals. A GOY sample (XYY) was also identified above the top left cluster. B-d identification and characterization of outliers of the PAR1 Bdev – mLRRy plot by representation of LRR and B allele

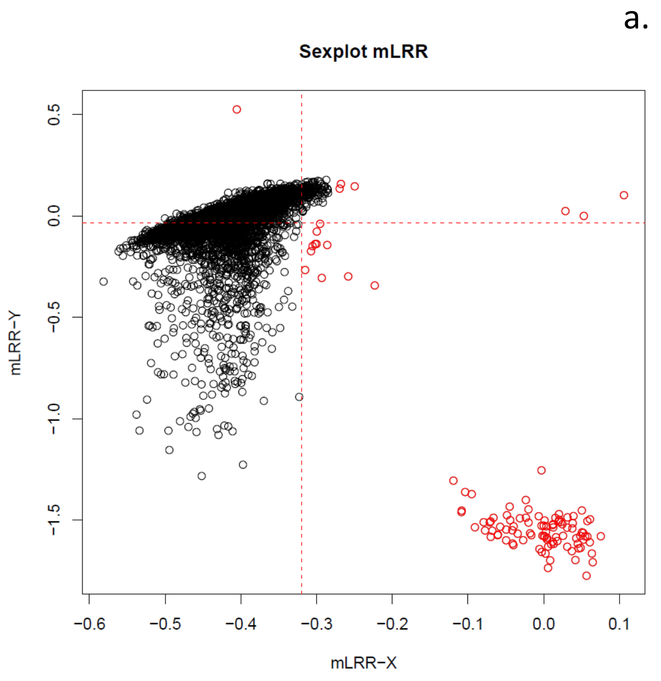

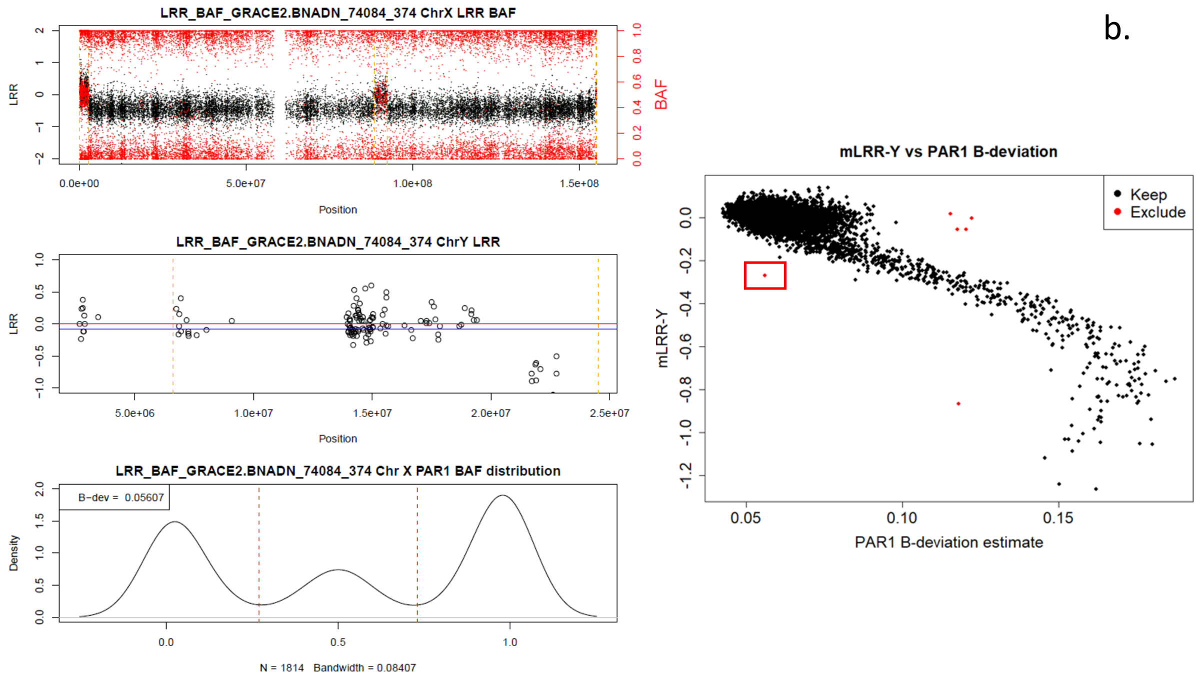

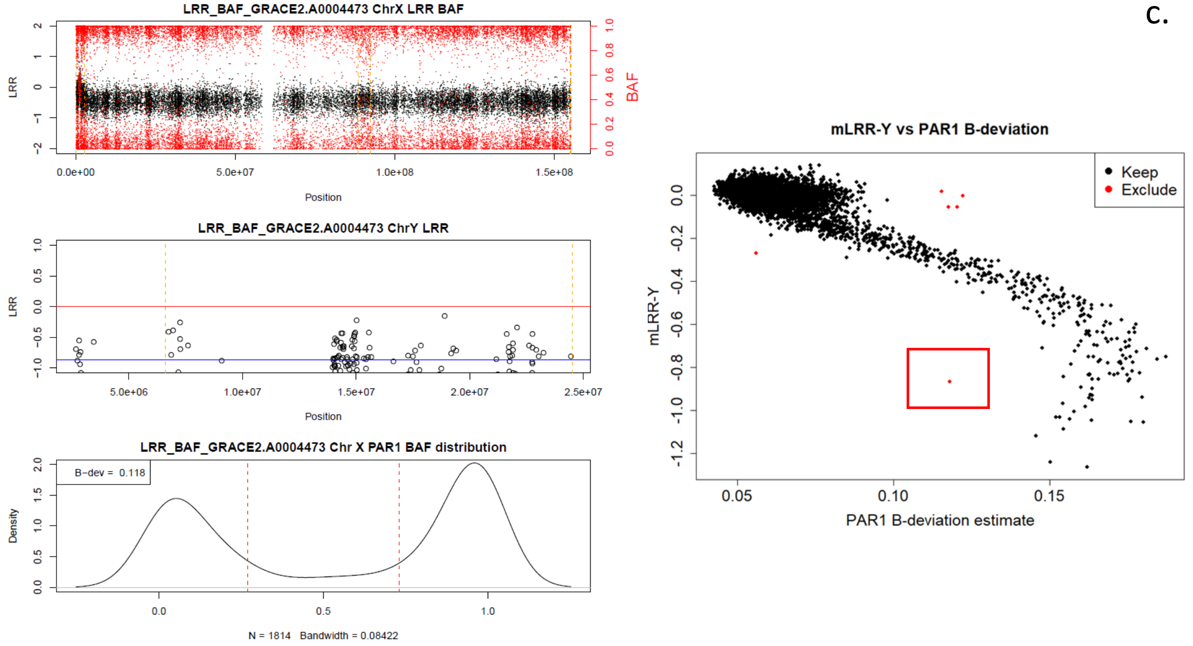

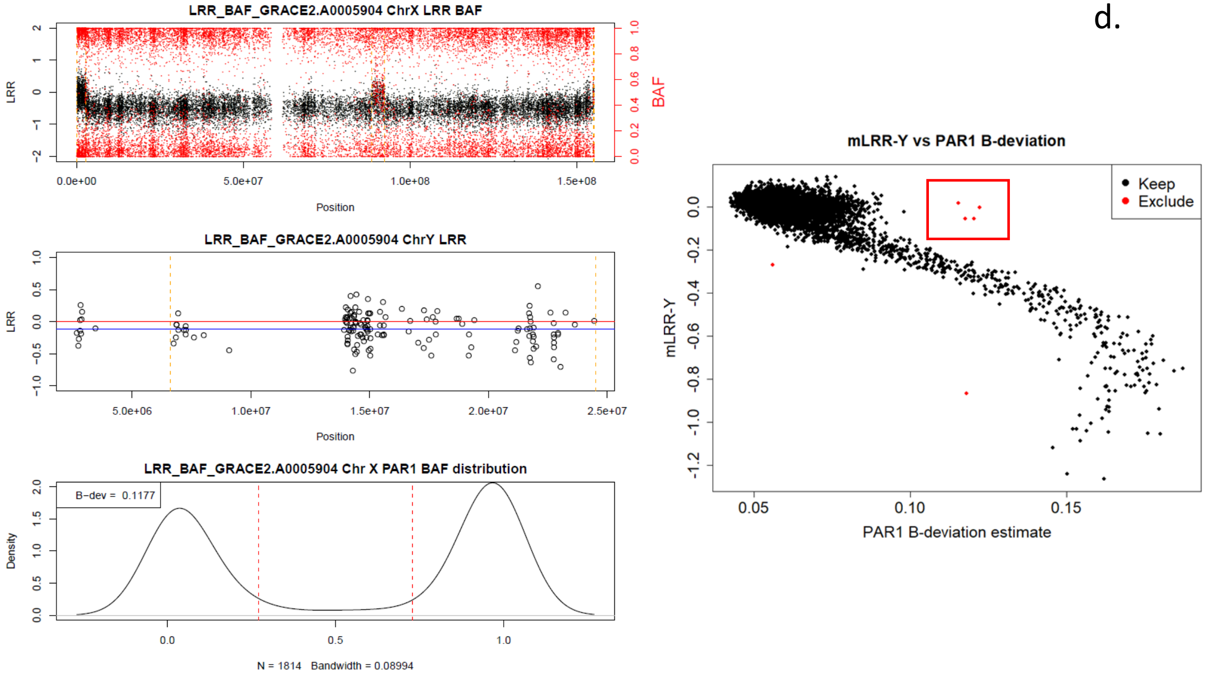

Supplement: Supplementary file 1 [file ijms-24-00898-s001.zip › Figure_S1.pdf]
