# Supplementary material for: Mendelian Randomisation Confirms the Role of Y-Chromosome Loss in Alzheimer’s Disease Aetiopathogenesis in Men
Source: Int J Mol Sci. 2023 Jan 4;24(2):898. doi: 10.3390/ijms24020898 (PMC9863537; doi:10.3390/ijms24020898)

Figure S2. Analysis of batch effect in mLOY calling.
We tested a linear model using batch and age at extraction as predictors of mLRR-Y (continous mLOY parameter).

	Beta	SE	P
**Batch**	$-6.02 \cdot 10^{-3}$	$5.17 \cdot 10^{-3}$	0.24
**Age**	$3.57 \cdot 10^{-3}$	$2.75 \cdot 10^{-4}$	$1.39 \cdot 10^{-37}$

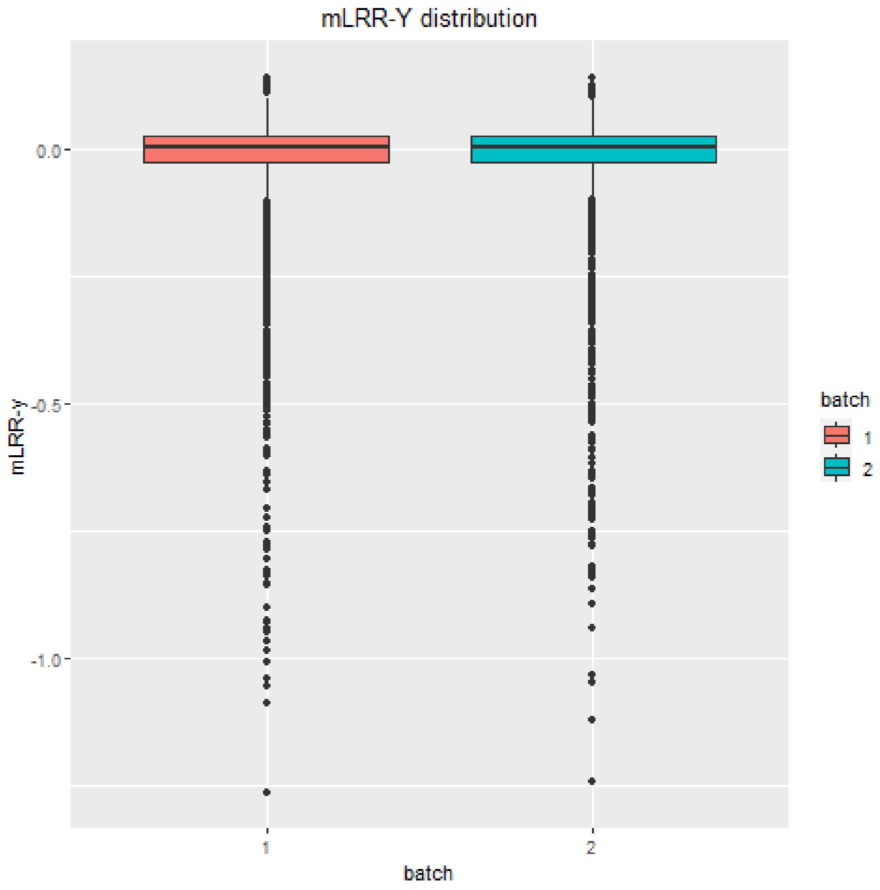

Supplement: Supplementary file 1 [file ijms-24-00898-s001.zip › Figure_S2.pdf]
