# Supplementary material for: Mendelian Randomisation Confirms the Role of Y-Chromosome Loss in Alzheimer’s Disease Aetiopathogenesis in Men
Source: Int J Mol Sci. 2023 Jan 4;24(2):898. doi: 10.3390/ijms24020898 (PMC9863537; doi:10.3390/ijms24020898)
Supplement: Supplementary file 1 [file ijms-24-00898-s001.zip › Figure_S3.pdf]

**Figure S3**. **Flow chart describing QC and filtering steps prior to case-control and disease progression analysis, depending on the use of mLOY phenotype or mloy-PRS as the independent variable.**

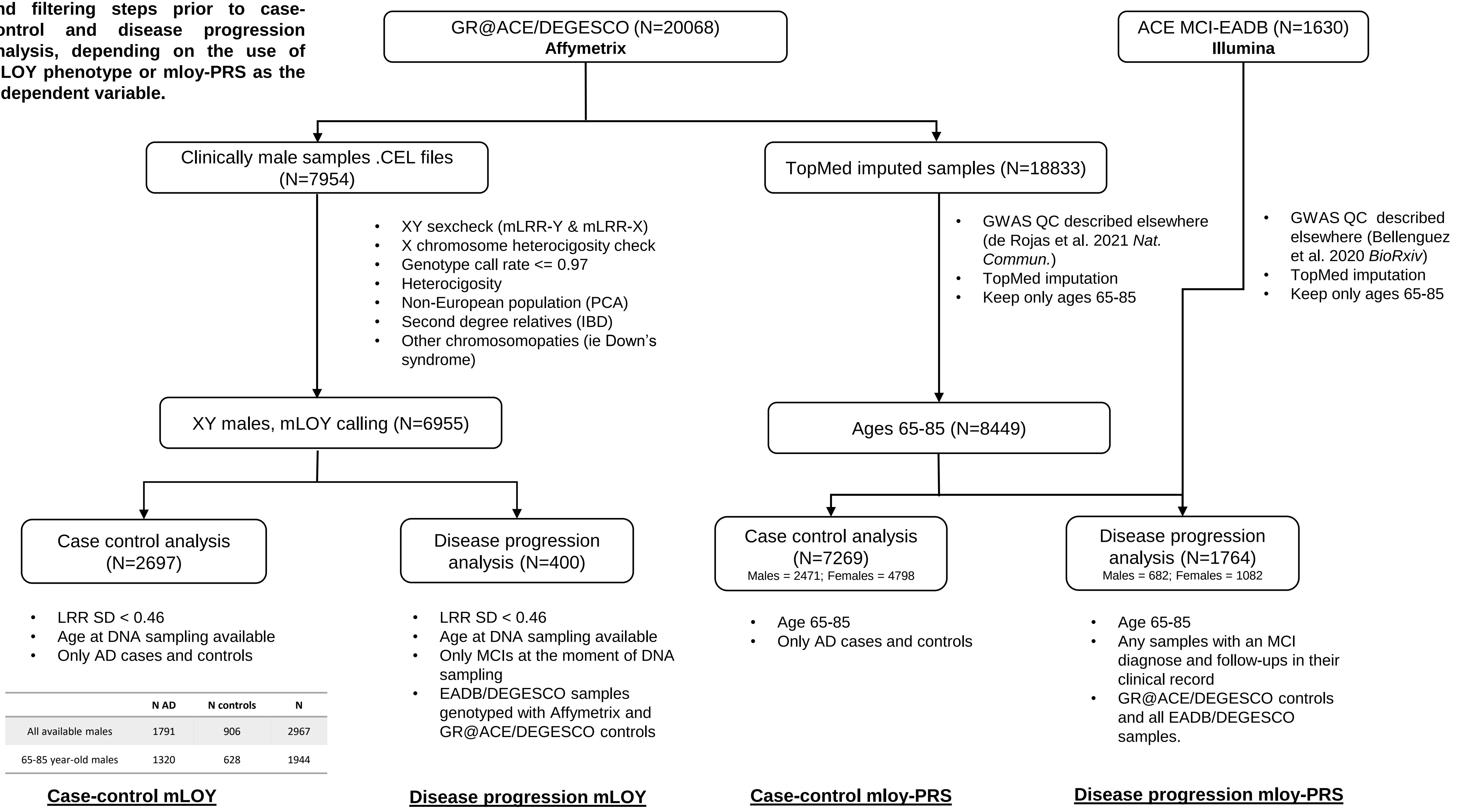


| | N AD | N controls | N |
|---|---|---|---|
| All available males | 1791 | 906 | 2967 |
| 65-85 year-old males | 1320 | 628 | 1944 |
