# Supplementary material for: Mendelian Randomisation Confirms the Role of Y-Chromosome Loss in Alzheimer’s Disease Aetiopathogenesis in Men
Source: Int J Mol Sci. 2023 Jan 4;24(2):898. doi: 10.3390/ijms24020898 (PMC9863537; doi:10.3390/ijms24020898)
Supplement: Supplementary file 1 [file ijms-24-00898-s001.zip › Figure_S4.pdf]

Figure S4. Validation and age independence of the mloy-PRS variable. a. PRS tertile distribution across the age spectrum. **b.** Linear regression of age (independent variable) with mLOY PRS (dependent variable) in the 65-85 age group.

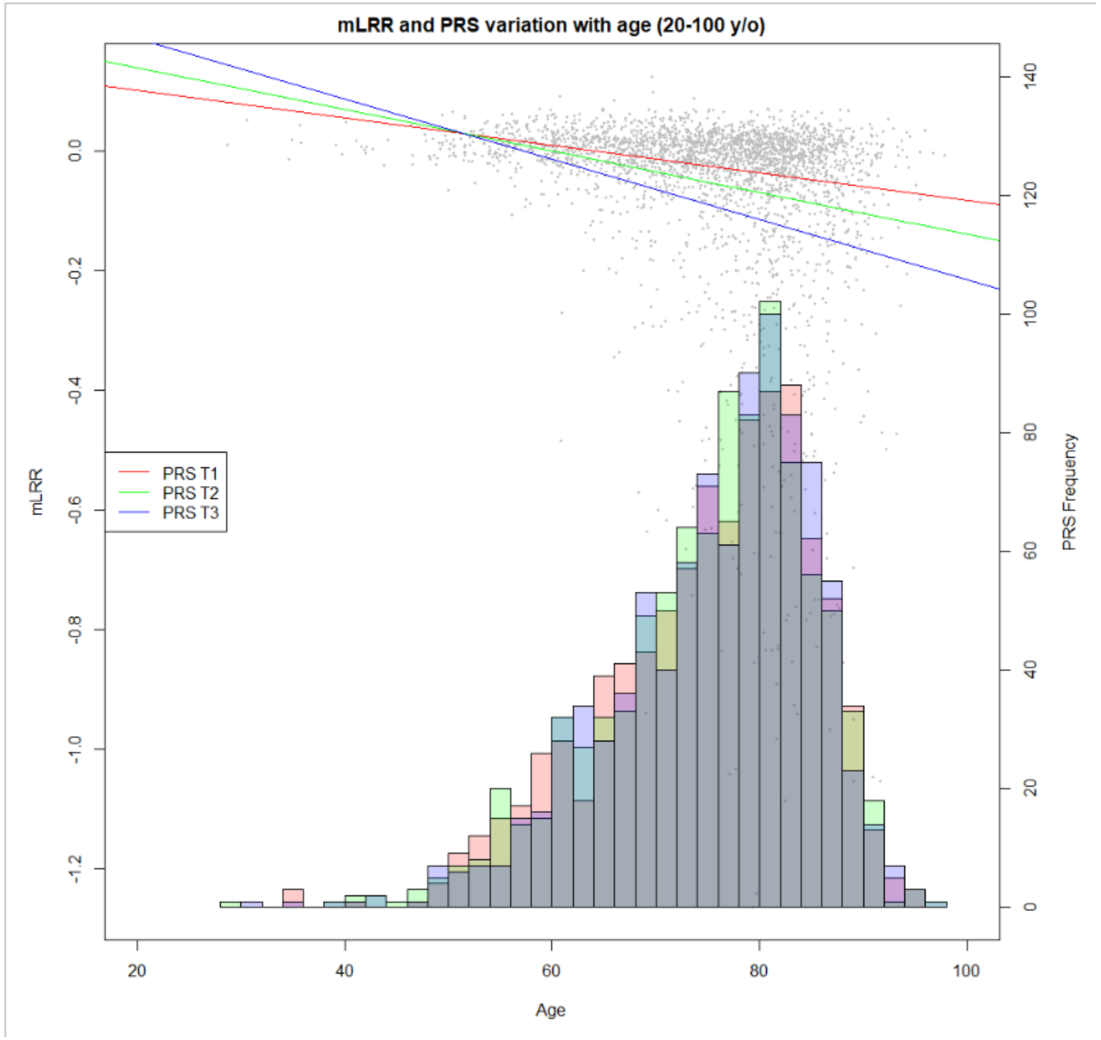

b.	Beta	SE	t	P	CI2.5	CI97.5
**Age**	0.00	0.02	0.13	0.90	-0.03	0.04
