# Supplementary material for: Mendelian Randomisation Confirms the Role of Y-Chromosome Loss in Alzheimer’s Disease Aetiopathogenesis in Men
Source: Int J Mol Sci. 2023 Jan 4;24(2):898. doi: 10.3390/ijms24020898 (PMC9863537; doi:10.3390/ijms24020898)

Figure S5. **mloy-PRS association with MCI progression to all-cause-dementia and AD-dementia.** Kaplan-Meier plots represent survival curves for individuals classified by mloy-PRS tertiles. Forest plots show the effect sizes of mloy-PRS, age, and *APOE* genotype in the models for conversion to dementia (**a**) or AD (**b**) in females and conversion to dementia (**c**) or AD (**d**) in males.

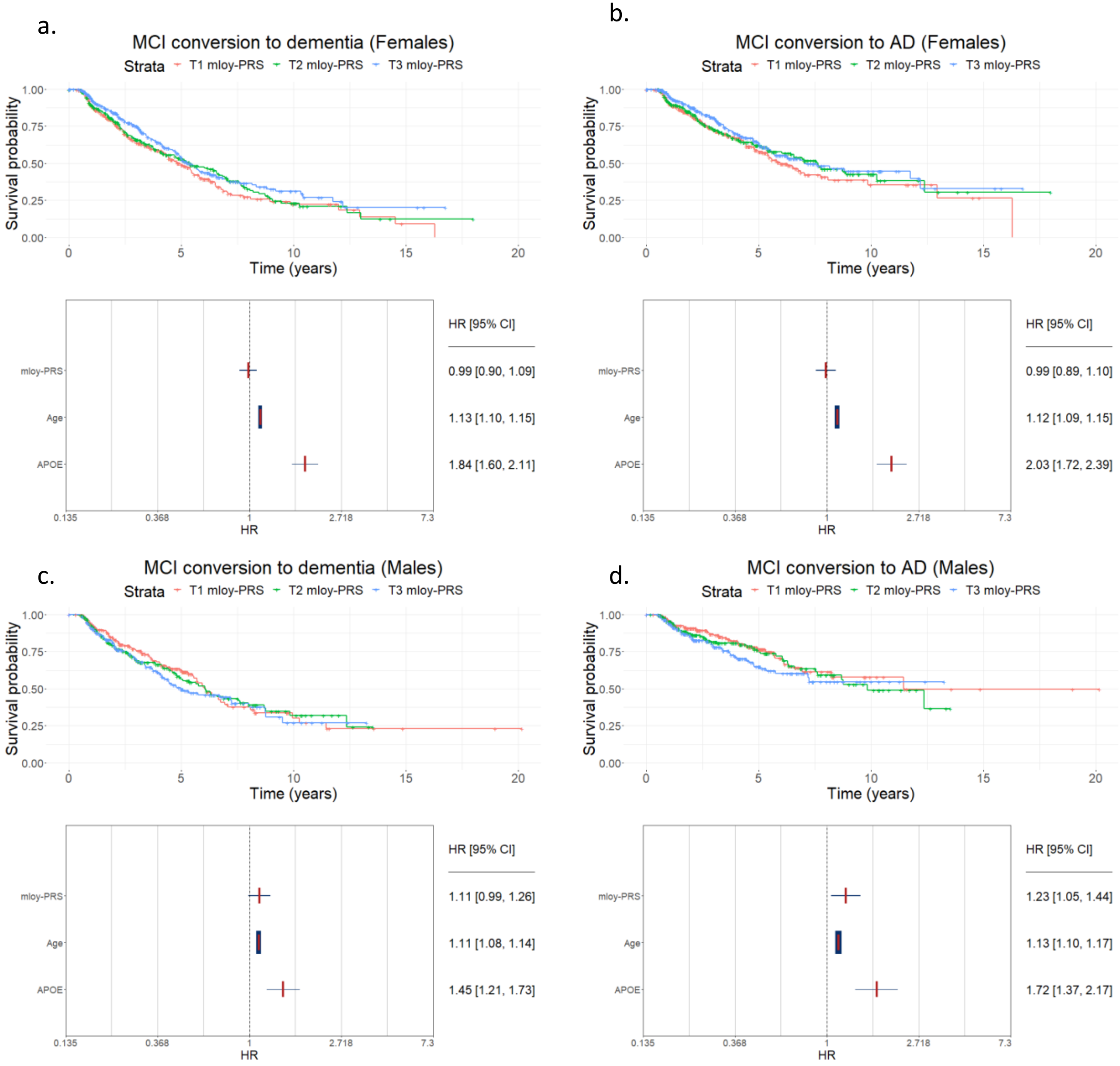

Supplement: Supplementary file 1 [file ijms-24-00898-s001.zip › Figure_S5.pdf]
