# Supplementary material for: Mendelian Randomisation Confirms the Role of Y-Chromosome Loss in Alzheimer’s Disease Aetiopathogenesis in Men
Source: Int J Mol Sci. 2023 Jan 4;24(2):898. doi: 10.3390/ijms24020898 (PMC9863537; doi:10.3390/ijms24020898)

Figure S6. Overview of mLRR-Y and PAR1-Bdev distributions in our dataset. a. Plot showing mLRR-Y and pseudoautosomal región 1 B-deviation (PAR1-Bdev) values for male samples in the GR@ACE/DEGESCO cohort. Red-colored dots represent samples displaying anomalies (Figure S2), which were discarded from further analysis. B-deviation was calculated as the mean BAF absolute deviation from 0.5 for heterozygous probes. Heterozygous probes were considered as those with a BAF in the range of 0.23–0.77. High correlation between mLRR-Y and PAR1-Bdev was observed for the majority of samples with a low to intermediate degree of mLOY (mLRR-Y ranging from -0.1 to -0.6). However, such correlation was lost at lower mLRR-Y values due to a decrease in the amount of probes with BAF values between the fixed thresholds. Thus, an accurate PAR1-Bdev measurement for samples with a high degree of mLOY would require using a dynamic window with more inclusive thresholds at lower mLRR-Y values in order to identify heterozygous probes. This phenomenon was easily observed when we compared PAR1 B allele frequency (PAR1 BAF) distributions in a sample with no LOY (**b**), moderate mLOY (**c**), or extreme mLOY (**d**).

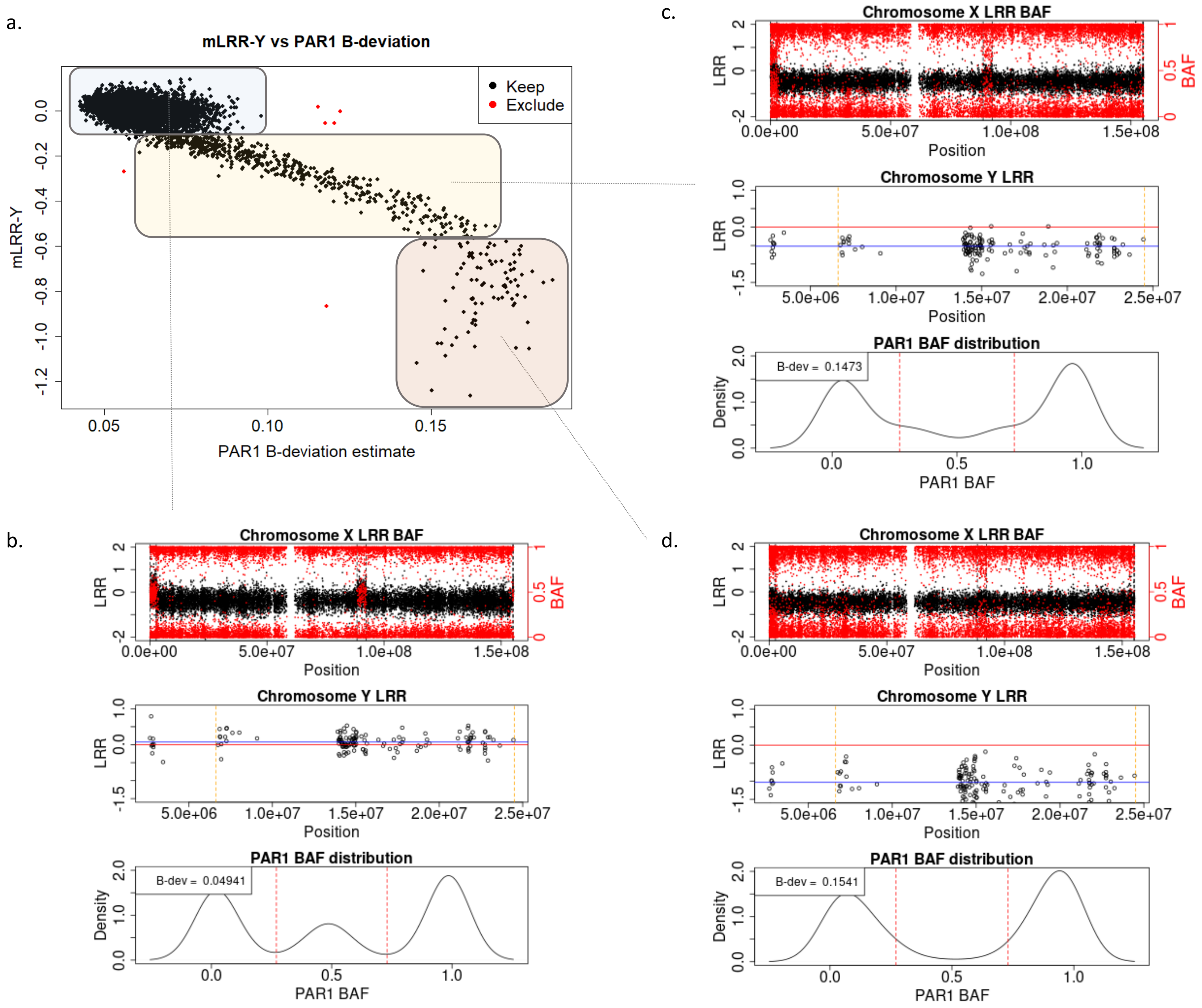

Supplement: Supplementary file 1 [file ijms-24-00898-s001.zip › Figure_S6.pdf]
