# Supplementary material for: Mendelian Randomisation Confirms the Role of Y-Chromosome Loss in Alzheimer’s Disease Aetiopathogenesis in Men
Source: Int J Mol Sci. 2023 Jan 4;24(2):898. doi: 10.3390/ijms24020898 (PMC9863537; doi:10.3390/ijms24020898)
Supplement: Supplementary file 1 [file ijms-24-00898-s001.zip › Figure_S7.pdf]

Figure S7. Association between PCs and conversion from MCI to dementia (PRS analysis). PC1 is highly associated to AD, but in fact represents genotyping chip/cohort ascertainment as shown. Thus, PC1 was not included in the models, as we already adjusted our data by genotyping chip/cohort ascertainment.

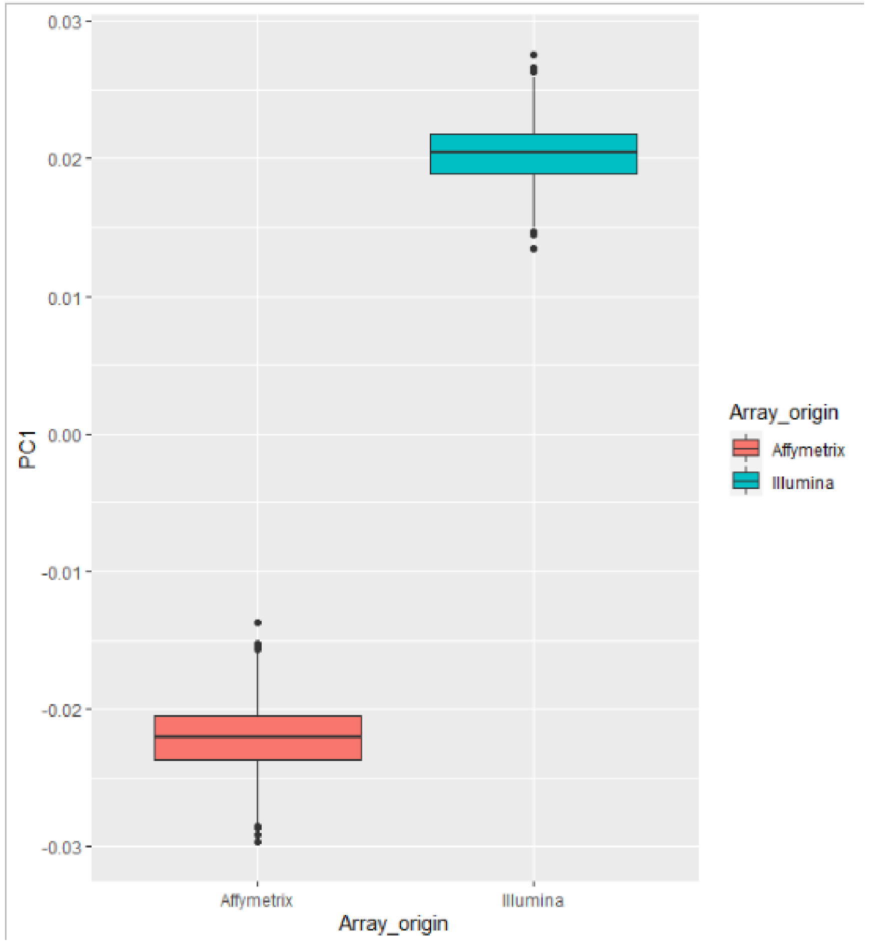

	coef	exp(coef)	se(coef)	z	Pr(>|z|)	2.5 %	97.5 %
**PC1**	-18.58	$8.50 \cdot 10^{-9}$	1.63	-11.38	**5.45**$\cdot 10^{-30}$	-21.78	-15.38
PC2	1.44	4.21	1.54	0.94	0.35	-1.57	4.45
PC3	-0.21	0.81	1.58	-0.13	0.90	-3.29	2.88
PC4	-1.60	0.20	1.58	-1.01	0.31	-4.69	1.49
PC5	-1.97	0.14	1.60	-1.23	0.22	-5.10	1.16
