# Supplementary material for: Mendelian Randomisation Confirms the Role of Y-Chromosome Loss in Alzheimer’s Disease Aetiopathogenesis in Men
Source: Int J Mol Sci. 2023 Jan 4;24(2):898. doi: 10.3390/ijms24020898 (PMC9863537; doi:10.3390/ijms24020898)
Supplement: Supplementary file 1 [file ijms-24-00898-s001.zip › Table_S1.pdf]

Table S1. Age and sample size of the case and control populations in the GR@ACE-DEGESCO cohort before and after age filtering. Only samples with available age at DNA extraction were considered. SD, Standard Deviation.

	N total	N group	Age total; Mean (SD)	Age groups; Mean (SD)	
All cases and controls	2697	906	75.5 (9.5)	68.7 (9.1)	Control
		1791		79.0 (7.7)	AD
Age 65-85	1944	624	76.4	73.5 (4.6)	Control
		1320		77.8 (4.8)	AD
