# Supplementary material for: Mendelian Randomisation Confirms the Role of Y-Chromosome Loss in Alzheimer’s Disease Aetiopathogenesis in Men
Source: Int J Mol Sci. 2023 Jan 4;24(2):898. doi: 10.3390/ijms24020898 (PMC9863537; doi:10.3390/ijms24020898)
Supplement: Supplementary file 1 [file ijms-24-00898-s001.zip › Table_S11.pdf]

Table S11. **Sample contribution by centre to the complete GR@ACE-DEGESCO cohort and different sub analysis selections.**

Center	Source of DNA	GR@ACE-DEGESCO	PRS case-control	mLOY case-control	mLOY case-control (age 65-85)
ACE Alzheimer Center Barcelona	Blood	8722	4926	2286	1552
Banco Nacional de ADN	Blood	3738	307		
Centro de Biología Molecular Severo Ochoa	Blood	23	21		
Fundación CIEN	Blood	1322	904	411	392
HRC	Blood	38	25		
Hospital Universitario Central de Asturias	Blood	195	148		
Hospital Universitario de Valme	Blood	1022	30		
IBIS	Blood	1005	445		
IRBLleida	Blood	230	160		
Mutua de Terrassa	Blood	247	153		
Universidad de Málaga	Saliva	408	78		
Hospital Universitario de Marqués de Valdecilla	Blood	298	72		
		INCLUSION CRITERIA:	• Age 65-85	• Age at DNA sampling available<br>• Males	• Age at DNA sampling 65-85<br>• Males
