# Supplementary material for: Mendelian Randomisation Confirms the Role of Y-Chromosome Loss in Alzheimer’s Disease Aetiopathogenesis in Men
Source: Int J Mol Sci. 2023 Jan 4;24(2):898. doi: 10.3390/ijms24020898 (PMC9863537; doi:10.3390/ijms24020898)
Supplement: Supplementary file 1 [file ijms-24-00898-s001.zip › Table_S12.pdf]

Table S12. Cox proportional-hazards model results for PCs association to conversion from MCI to dementia in prospective individuals with MCI from the GR@ACE-DEGESCO cohort. HR, hazard ratio; SE, standard error, CI2.5, 2.5% quantile; CI97.5, 97.5% quantile.

	log(HR)	HR	SE	Z	P	CI2.5	CI97.5
PC1	-4.28	0.01	3.92	-1.09	0.27	-11.96	3.39
PC2	-1.38	0.25	1.46	-0.94	0.35	-4.24	1.49
PC3	-0.48	0.62	1.65	-0.29	0.77	-3.72	2.76
PC4	-0.36	0.70	1.48	-0.24	0.81	-3.25	2.54
