# Supplementary material for: Mendelian Randomisation Confirms the Role of Y-Chromosome Loss in Alzheimer’s Disease Aetiopathogenesis in Men
Source: Int J Mol Sci. 2023 Jan 4;24(2):898. doi: 10.3390/ijms24020898 (PMC9863537; doi:10.3390/ijms24020898)
Supplement: Supplementary file 1 [file ijms-24-00898-s001.zip › Table_S2.pdf]

Table S2. ANCOVA adjusted by age at sampling and APOE genotype, using the case-control variable as an independent variable and mLRR-Y as the dependent variable. Only individuals with available age at DNA sampling information were included in the analysis. Df, degrees of freedom; Sum Sq, sum of squares; Mean Sq, mean squares.

	Df	Sum Sq	Mean Sq	F value	Pr(>F)
Case-control (1/0)	1	1.54	1.54	67.99	2.54E-16
Age at sampling	1	1.84	1.84	81.22	3.73E-19
APOE	1	0.09	0.09	3.86	4.94E-02
Residuals	2693	61.14	0.02		
