# Supplementary material for: Mendelian Randomisation Confirms the Role of Y-Chromosome Loss in Alzheimer’s Disease Aetiopathogenesis in Men
Source: Int J Mol Sci. 2023 Jan 4;24(2):898. doi: 10.3390/ijms24020898 (PMC9863537; doi:10.3390/ijms24020898)
Supplement: Supplementary file 1 [file ijms-24-00898-s001.zip › Table_S3.pdf]

Table S3. Logistic regression results for PC association with the AD case-control variable in the mLOY/mLRR-Y case-control analysis. Only individuals with age at extraction 65-85 years old were included in the models. OR, odds ratio; SE, standard error; CI2.5, 2.5% quantile; CI97.5, 97.5% quantile.

	**log(OR)**	**SE**	**Z**	**P**	**CI2.5**	**CI97.5**
**PC1**	-5.93	2.81	-2.11	0.03	-11.44	-0.41
**PC2**	-5.75	2.88	-2.00	0.05	-11.40	-0.11
**PC3**	3.78	3.04	1.24	0.21	-2.17	9.75
**PC4**	-2.61	3.69	-0.71	0.48	-9.85	4.62
