# Supplementary material for: Mendelian Randomisation Confirms the Role of Y-Chromosome Loss in Alzheimer’s Disease Aetiopathogenesis in Men
Source: Int J Mol Sci. 2023 Jan 4;24(2):898. doi: 10.3390/ijms24020898 (PMC9863537; doi:10.3390/ijms24020898)
Supplement: Supplementary file 1 [file ijms-24-00898-s001.zip › Table_S5.pdf]

Table S5. Logistic regression results for mloy-PRS, age at sampling and APOE genotype (independent variables) versus mLOY status (dependent variable). Only males aged 65-85 were included in the models. OR, odds ratio; SE, standard error; CI2.5, 2.5% quantile; CI97.5, 97.5% quantile.

	OR	SE	Z	p	CI2.5	CI97.5
mLOY PRS	1.80	1.07	9.18	$4.22 \cdot 10^{-20}$	0.46	0.72
Age at sampling	1.08	1.01	6.57	$5.07 \cdot 10^{-11}$	0.06	0.10
APOE	0.88	1.09	-1.45	0.15	-0.30	0.04
