# Supplementary material for: Mendelian Randomisation Confirms the Role of Y-Chromosome Loss in Alzheimer’s Disease Aetiopathogenesis in Men
Source: Int J Mol Sci. 2023 Jan 4;24(2):898. doi: 10.3390/ijms24020898 (PMC9863537; doi:10.3390/ijms24020898)
Supplement: Supplementary file 1 [file ijms-24-00898-s001.zip › Table_S6.pdf]

Table S6. Logistic regression results for PCs association to AD case-control variable in the mLOY PRS case-control analysis. Only individuals aged 65-85 were included in the models. OR, odds ratio; SE, standard error; CI2.5, 2.5% quantile; CI97.5, 97.5% quantile.

ALL (MALES + FEMALES)	log(OR)	SE	Z	P	CI2.5	CI97.5
PC1	-0.89	2.32	-0.38	7.02E-01	-5.46	3.65
PC2	-23.80	2.37	-10.05	8.74E-24	-28.46	-19.18
PC3	-18.71	2.41	-7.77	7.68E-15	-23.45	-14.01
PC4	0.32	2.34	0.14	8.93E-01	-4.27	4.90
**MALES**	**log(OR)**	**SE**	**z**	**P**	**CI2.5**	**CI97.5**
PC1	-1.56	2.38	-0.65	5.12E-01	-6.21	3.14
PC2	-18.38	2.46	-7.47	7.78E-14	-23.23	-13.59
PC3	8.62	2.43	3.55	3.83E-04	3.87	13.39
PC4	-1.91	3.09	-0.62	5.36E-01	-7.96	4.14
**FEMALES**	**log(OR)**	**SE**	**z**	**P**	**CI2.5**	**CI97.5**
PC1	1.71	2.33	0.74	4.62E-01	-2.83	6.31
PC2	-14.56	2.37	-6.14	8.07E-10	-19.22	-9.93
PC3	12.38	2.37	5.23	1.72E-07	7.75	17.03
PC4	3.19	2.32	1.37	1.70E-01	-1.36	7.74
